# Provider perspectives on chronic kidney disease diagnosis delivery 

**DOI:** 10.5414/CN109153

**Published:** 2017-10-23

**Authors:** Hannah Tiu, Angela Fagerlin, Meghan Roney, Eve Kerr, Akinlolu Ojo, Ed Rothman, Julie Wright Nunes

**Affiliations:** 1 Department of Medicine, University of Arizona Health Sciences, Tucson, AZ,; 2 Department of Population Health Sciences, Salt Lake City,; 3 Salt Lake City VA Center for Informatics Decision Enhancement and Surveillance (IDEAS), University of Utah, Salt Lake City, UT,; 4 Center for Bioethics and Social Sciences in Medicine, University of Michigan,; 5 Veterans Affairs Healthcare System and Ann Arbor Center for Clinical Management Research,; 6 Department of Statistics, University of Michigan, and; 7 Department of Internal Medicine, University of Michigan, Ann Arbor, MI, USA

**Keywords:** provider perspectives, chronic kidney disease, mixed-methods, qualitative research, quality improvement

## Abstract

Aims: Lack of clear provider communication has been suggested as a reason for low patient awareness of their chronic kidney disease (CKD) diagnosis. Using quality improvement methods, we performed one-on-one provider interviews about CKD diagnosis delivery. Materials and methods: Interviews were audio-recorded, transcribed, and examined using mixed methods. We used thematic analysis to code and analyze transcripts, and Fisher’s exact test to examine differences comparing nephrologist and primary care provider (PCP) perspectives. Results: 24 providers completed interviews (18 nephrologists, 6 PCPs). Four themes emerged (N = 260 statements): 1) perspectives informing patients about CKD diagnosis (37 statements), 2) timing of diagnosis messaging (38 statements), 3) language used to convey diagnosis (42 statements), and 4) challenges in diagnosis delivery (143 statements). Most agreed that patients should be informed of their CKD (87.5%), but only 76% believed that communication should occur early. Terminology was not unified; half of nephrology providers used the term “Chronic Kidney Disease” to explain diagnosis. No PCPs used this terminology. Challenges to CKD diagnosis delivery included: Kidney disease is perceived as difficult to explain, lack of provider time, lack of patient symptoms, patient denial of disease, and low public awareness of CKD. Conclusions: Providers’ views on informing patients of their CKD diagnosis were not unified, in particular with respect to timing and terminology of diagnosis delivery. More work is needed to address barriers to efficiently and effectively convey CKD diagnosis information.

## Introduction 

Chronic kidney disease (CKD) is a serious public health issue. One in 10 Americans has kidney disease according to the Centers for Disease Control and Prevention [1]. This is especially problematic as people who have CKD are at high risk for mortality [2] and require care that is often associated with significant economic burdens [3, 4]. Encouragingly, when patients are actively engaged in care, they can often reverse disease-related complications and achieve great strides optimizing their health despite having a chronic disease [5, 6, 7, 8]. 

Informing patients about their kidney disease seems intuitively to be an important initial component of engaging patients in care [9]. It is hard to imagine a patient fully collaborating with their provider to preserve existing kidney function without first understanding they have a chronic kidney condition. There is data showing that most patients with CKD are not aware of their disease [10, 11]. However, when patients are more “aware” and educated about CKD, they realize clinical benefits. For instance, the Kidney Early Evaluation Program (KEEP) focused on increasing awareness about kidney disease among high-risk individuals. Research shows KEEP patient participants received earlier CKD care by a nephrologist, were more apt to seek home dialysis modalities, and were more likely to receive transplantation once at end-stages of kidney disease compared to nonparticipants [12]. 

Inadequate recognition of, and counseling patients about CKD from providers have been suggested as two reasons for a lack of patient diagnosis awareness [11]. Initial data from small focus groups supports this, showing primary care providers (PCPs) do perceive barriers to educating patients about kidney disease [13, 14]. A recent finding by Wright et al. [15] shows that patients want to be informed about their CKD diagnosis and that they want to know about CKD diagnosis information early, despite their own fears of advancing disease, dialysis, and death. However, there is little data describing provider perspectives across practice settings on how and when a CKD diagnosis should be delivered. For example, when the topic is introduced, what language do providers use? When do providers feel patients should be informed of their CKD diagnosis? Do providers uniformly agree that patients should be informed in the first place? These answers cannot be assumed. Exploring provider’s perspectives on CKD diagnosis delivery, as the initiating point of further CKD discussion, could help elucidate reasons for lack of patient awareness and existing patient perceptions that diagnostic information is relayed too late [15]. 

We designed a study to explore perspectives about how and when providers choose to deliver a CKD diagnosis to patients. We did this to identify potential areas where support may be needed at the provider level to optimize diagnosis communication and to inform future interventions to increase patient CKD awareness. We planned to incorporate insights into own ongoing research examining the impact of CKD educational tools designed for use at the patient/provider interface. The study included methods to examine the information both qualitatively and, where applicable, quantitatively, i.e., using mixed methods. 

## Materials and methods 

### Study design 

We aligned reporting with consolidated criteria for reporting qualitative research (COREQ) [16]. This research is part of an ongoing qualitative study in CKD education [15]. Interviews were conducted using open-ended but predefined questions based on two CQI (continuous quality improvement) methods; namely cause/effect analysis and quality function deployment (QFD) [17, 18]. Cause/effect analysis is a diagram-based approach for thinking through all of the possible causes of a problem in order to address them [17]. QFD is a structured approach to defining “customer” needs and translating them into specific plans to meet those needs [19]. The reason the questions were designed around these methods is because these methods offered a systematic way to think about and break down all of the potential “problems” in the current processes of patient-provider communication. Specifically, we created questions about what could be potential negative “effects” related to low patient awareness and knowledge about CKD, along with potential causes, and then designed questions to gain insights into these areas. For example, one negative “effect” is low patient CKD knowledge, so we asked providers questions about the causes for this, e.g., “what are the causes for low patient disease knowledge?” In the interviews, we aligned questions to be specific to potential provider-related causes; these questions are shown in Table 1. 

### Participants and setting 

The provider population included physicians from nephrology, family medicine, and internal medicine clinics affiliated within one academic center. Providers were invited to participate through announcements at faculty meetings and email. Nonlicensed medical trainees were excluded. The Institutional Review Board at the University of Michigan approved all study procedures prior to enrollment. One-on-one structured interviews were conducted in person, in predefined private rooms, by trained research personnel. Written informed consent was obtained prior to research activity. 

### Data collection 

Provider characteristics were collected including age, sex, race, and ethnicity (specifically we asked if patients were of Hispanic, Latino, or Middle Eastern origin). We also asked when providers completed medical school training, a measure we called “years in practice”. Interviews were audio-recorded and transcribed verbatim. Participants were enrolled until thematic saturation was reached whereby additional interviews did not provide new information [20, 21]. 

### Data analysis 

Interview transcripts were imported into Dedoose™ (http://dedoose.com/). Dedoose is a software program used for coding and analysis of qualitative data and allows further descriptive analyses by integrating transcribed data with demographic and other quantitative measures. We used thematic analysis to qualitatively examine the data. Thematic analysis involves grouping the data into themes that will help answer the research questions [22]. Themes can be directly related to the research questions or may naturally emerge from the study. In our study, the process was iterative and included elements of both approaches. Study team members listed themes as they evolved – many aligned with study questions. After interviews were completed, the study team finalized thematic groups and categorized the data into these groupings by “coding” the transcripts. The coding was done in duplicate, independently by two members of the study team. The two coders then met to review coding results, identify differences, and resolving differences by consensus. 

Statement frequencies were tallied and presented as n (%). Continuous variables were presented at mean (SD). Questions lending to dichotomous responses were identified, and these responses were then entered into a spreadsheet for analysis along with provider characteristics (Microsoft Excel version 2013). We used Fisher’s exact test to examine for differences in dichotomous responses between groups of providers based on medical discipline (nephrologist or PCP) and Wilcoxon rank-sum to determine if there were differences based on provider years in practice. A p-value < 0.05 was considered statistically significant. All statistical analyses were performed using IBM SPSS standard version. 

## Results 

We interviewed 25 providers from January to October 2014. There were 18 nephrologists and 7 PCPs. One audio-file was corrupted and hence not included in the final analysis of 18 nephrologists and 6 PCPs. The mean (SD) age of participants was 46 ± 9 years, 36% were women, and 67% were white. These and additional baseline characteristics are reported in Table 2. 

Four themes emerged with 260 total statements about CKD diagnosis delivery. Major themes were: 1) perspectives on informing patients about their CKD diagnosis, 2) timing of diagnosis delivery, 3) language used to convey diagnosis, and 4) challenges in diagnosis delivery. The majority of statements reflected challenges to diagnosis delivery. Below are descriptions of each major theme with representative statements and their attributions (Neph = nephrologist, PCP = primary care provider). 

### Perspectives on informing patients about their CKD diagnosis (37 statements) 

This theme encompasses provider perspectives on whether or not patients should be told they have chronic kidney disease. Although the majority (n = 21, 87.5%) felt that patients should be informed about their CKD, some providers (n = 3, 12.5%) felt that informing patients was conditional, e.g., depending on CKD stage, whether the patient is already anxious about another illness, and/or whether the provider felt CKD itself could negatively impact the patient’s life. There was no statistically-significant difference comparing responses between medical disciplines broken down by nephrology vs. primary care (66.7% of nephrologists vs. 94% PCPs, p = 0.14) (Table 3). However, we found a significant difference in that providers with a higher number of years in practice felt informing patients was conditional and based upon other factors. The mean (SD) years in practice in providers who were hesitant to inform patients was 35.6 (4.9) years versus 19.1 (7.9) years in those who unequivocally felt we should inform patients (p = 0.01). Below are two representative statements reflecting different provider perspectives. 

*“For me I really want to impress upon them (patients) that there is this disease and they need to be careful to avoid nephrotoxic agents and why it’s very important for blood pressure control.” (Neph18)*

*“So it depends … I think the question of when do I tell them really has more to do with how far do I believe they are away from the kidney disease impacting the quality or length of their life. So if I believe that … it’s going to shorten their life, then I will discuss it with them. If I believe that it will not shorten their life, then my discussion with them is ... you still have to say something … but I will word it very differently, and I will pass it off as no big deal.” (PCP1)*

### Timing of diagnosis messaging (38 statements) 

This theme reflects statements about timing of when to inform patients of their CKD diagnosis. While most providers (78%) felt patients should be informed early in the spectrum of CKD, some expressed hesitancy (24%). Those who were hesitant cited other factors they felt needed to be considered first, including: a patient’s overall health status, established rapport between provider and patient, and trajectory of worsening kidney function (whether providers thought CKD could get worse). We identified a trend comparing how PCPs and nephrologists felt about this topic (87.5% of nephrologists felt patients should be told early vs. only 40% of PCPs, p = 0.06). There was not a statistically-significant difference comparing years in practice. Below are representative statements reflecting different perspectives. 

*“Earlier is better because there are things that we can do to slow progression, so that we can talk about all of these things. They can understand so it‘s not a huge shock later if they‘ve done everything well and their kidneys get worse.” (Neph4)*

*“Generally I do it when a person’s creatinine is rising or their creatinine is above 1.5 … (but) ‘it depends’ is going to be the answer here, because you really have to think about the patient, how many other health issues they‘re up against, where their kidney disease falls in the big picture.” (PCP6)*

### Language used to convey diagnosis (42 statements) 

Specific terminology providers use to tell patients about their CKD diagnosis fell under this theme. Most providers chose not to use the term “chronic kidney disease”. Alternative terms included “low kidney function”, “decreased kidney function”, or expression of function as a percentage, e.g., “40% kidney function”. Comparing nephrologists to PCPs, no PCP used the term “chronic kidney disease”, and nephrologists were divided (0% of PCPs used the term “chronic kidney disease” vs. 56.3% of nephrologists, p = 0.05) (Table 3). No differences in years of experience were observed comparing providers using the term “CKD” to those who did not. Below are representative statements reflecting different perspectives: 

*“I tell them what it is. And what it’s called and how it’s staged and give them some idea of where they are at. In my day-to-day interactions I think, that’s very key.” (Neph12)*

*“I’ll say kidney dysfunction … I do feel like early on when somebody has early stage 3 CKD that to use the word disease can come across a little bit strong. So I do use the term, but I probably use “kidney dysfunction” a little bit more.” (Neph3)*

*“I guess I use “renal insufficiency”. But I don‘t know (if) that one is easier for a patient to hear.”(PCP3)*

### Challenges in diagnosis delivery (143 statements) 

Providers described the many challenges they faced when delivering a CKD diagnosis. Most common was that kidney disease is perceived as a very difficult concept to explain (n = 6, 28.2%). Interestingly, this was noted by nephrologists as well as PCPs. Other cited barriers included lack of patient symptoms to provide patients with clinical queues about their illness, patient denial or fear of having a chronic condition, and low public awareness of CKD – leading to many patients not knowing that this disease exists and could affect them. Others mentioned as barriers: a lack of provider time, the perception that CKD is not a distinct disease, patients’ differing levels of education, providers wanting to avoid instilling fear in patients, and lack of clear guidelines about how to discuss or even monitor CKD. 

*“The GFR I think … just explaining the GFR to them … is very difficult. What it means and … what your GFR is and what it is in relation to normal. I think that’s the hardest to explain.” (PCP4)*

*“Ah I would say that, one of the things is that most of the time this is not something the patients are aware of or feel any differently about … So basically it’s a silent disease, at least until the late stages and so you’re basically telling them about major changes that are coming.” (PCP6)*

*“… I think one of the things I had feedback from patients is that they feel that the nephrologists are, um, too scary, that they spend time about, oh well … it’s this long until you’ll probably be on dialysis. And patients do not want to hear that.” (PCP1)*

*“I am not given the time to take time (for) their complex medical issues and at the same time navigate through the system … often I’m not even … fully aware of when things are scheduled or what the exact resources are, so unless … the system provides some support for that … it’s a slower and less complete process than it might otherwise be.” (Neph14)*

## Discussion 

Our study found that most providers agree patients should be informed of their CKD diagnosis, however, those who have been in practice longer are more hesitant about informing patients. As a collective group, the providers felt patients should be told about CKD early in the disease course. However, this reflected mostly views of the nephrologists because 60% of PCPs were hesitant to do so, saying that ‘it depends’ on other factors. The term “chronic kidney disease” was not universally accepted for use by PCPs or nephrologists when delivering a CKD diagnosis to patients. Lastly, both PCPs and nephrologists cited many challenges to discussing CKD with patients including an inherent complexity in the diagnosis and its meaning. 

It was interesting that some providers expressed hesitancy in informing patients about their CKD diagnosis. Concerns about this focused on wanting to avoid anxiety or stress in patients. These thoughts echo opinions brought up by some nephrology researchers, as featured recently in the lay press [23]. Specifically, debate was highlighted about whether decreases in glomerular filtration rates should be defined the same in an elderly population as in a younger population. While some have argued the benefits to “diagnosis” even at older ages, others are not convinced it always merits distinction as a unique and chronic disease [23, 24]. This may explain why some providers in our study were also hesitant to disclose a diagnosis – as some stated they were unclear whether it would impact outcomes later on. 

However, recent work eliciting patient perspectives about receiving a CKD diagnosis uniformly shows that patients want to know diagnosis information at the earliest point it is identified by providers. In fact, patients often expressed frustration and even anger at not being told earlier, and at a point when they perceived they could have done something to prevent their CKD from getting worse [15]. Patients also felt that when information about diagnosis is delivered, terms providers used were not uniform or consistent, and they needed to be more clear for the benefit of the patient [15]. Knowing this could help providers overcome hesitancy about discussing a diagnosis. However, just as important as delivering information, is how to deliver it. Giving providers tools to optimize efficient delivery of diagnostic information or “bad” news into visits could help. 

A body of work in the oncologic literature suggests a framework for how to approach diagnosis communication sensitively and efficiently with patients. Admittedly, oncology is unique from CKD in many important ways. But a framework for handling sensitive conversations seems universal. Oncotalk^®^ (http://depts.washington.edu/oncotalk) provides one example [25], with online learning modules and videos that emphasize four fundamental principles for facilitating patient-provider communication: 1) finding out what patients know about their diagnosis as a first step, 2) identifying, acknowledging, and redirecting when conversations get off track, 3) responding to patients’ emotions after they hear about a “new” diagnosis, and 4) assessing patients’ current coping styles to use when responding to their emotions [25]. These principles are used with success in oncology [26, 27] and were also recently tested for use in palliative-care nephrology training [28]. Nephrology fellows who went through training reported higher self-efficacy and confidence approaching difficult conversations with patients. A part of this training included an emphasis on using an “ask-tell-ask” approach – asking what the patient understands about their condition, telling the patient about it, then reassessing for understanding [27]. These principles could be used by both nephrologists and PCPs delivering a CKD diagnosis; the framework provides a way to first check on what the patient knows as his/her “baseline” knowledge, and then a way to communicate more with them individually, to place their diagnosis in a proper context, and address their emotional needs as well. 

Interestingly, the term “chronic kidney disease” was not universally accepted for use by PCPs or nephrologists. Reasons cited were that it is a difficult term for patients to understand and that the concept of CKD is inherently difficult to explain – a view shared by both PCPs and nephrologists. Because one contributing factor mentioned was low public awareness, perhaps looking to programs in other chronic diseases that promote a baseline understanding in the general public [29] could inspire similar sustainable efforts within nephrology. Recent literature shows there is variation in patients’ awareness of CKD depending on how they are asked [30]. Until medical professionals can communicate a diagnosis clearly and consistently, it seems unlikely that we can make large gains to improve patient awareness of CKD across the continuum of their care [31]. 

It was interesting that many barriers cited to “educating” patients about CKD were shared by PCPs and nephrologists. Even lack of time to educate patients about CKD was cited as a challenge by nephrologists – who it seems, would have the most time to do just that. Our study did not explore why nephrologists had this view, and it should be examined in more detail in the future. But it does suggest that “educating” patients about their diagnosis may be perceived as different and perhaps secondary to other activities performed during the patient encounter. 

The United States National Kidney Disease Education Program (NKDEP) has developed several educational resources that could help providers efficiently deliver a CKD diagnosis to patients [32]. One NKDEP worksheet with slight modifications was pilot-tested with 155 patients seen in a nephrology practice and shown to improve patient-centered outcomes, despite taking only 1 – 2 minutes to review [33]. More work is needed to expand our understanding on how tools like this can be effectively used and sustained not only in nephrology but in primary care as well. 

While this study provides valuable insight into how healthcare providers think about communicating a new CKD diagnosis, it has limitations. For example, qualitative research can be subject to biases. We limited this by gathering a research team composed of individuals with multidisciplinary specialties, including areas outside of nephrology, who were less likely to have preconceptions about this research. Setting up advanced predefined questions and scripts for interviews also assisted in structuring and standardizing interviews. Another limitation is that the small sample size of providers affiliated with clinics from one academic medical center may limit generalizability. We attempted to maximize generalizability by including both PCPs and nephrologists and continued until thematic saturation was achieved, but there may be opportunity to gain more insights from a larger population in the future – especially with respect to getting more PCP perspectives, which were in the minority in this study. Despite our efforts during the interview to have each question answered explicitly, if providers chose not to, their response could not be included in the quantitative comparisons performed for some of the questions, which is why not all comparisons in Table 3 include a total N = 24 providers (Table 3). However, our study is unique in offering perspectives from both nephrologists and PCPs and gaining important perspectives qualitatively and quantitatively in key areas of patient-provider communication. 

There are several important implications of this study. Although most providers do feel patients should be informed of their CKD diagnosis, some are hesitant, and there is not unity on how or when to do it. This may account for lack of patient CKD awareness repeatedly identified through U.S. National Health and Nutrition Examination Survey (NHANES) through the years, even though most participants report having regularly scheduled medical follow-up [10, 11]. It is clear that all providers and especially PCPs want and need additional resources to help with diagnosis messaging. Merging tools that help predict patients at risk of CKD progression [34] with existing education resources could help tailor diagnosis messaging and education across the spectrum of CKD severity. 

In conclusion, we identified important perspectives from providers about how they deliver a diagnosis of CKD, and that there is hesitancy related to diagnosis messaging that may translate to lower diagnosis awareness and knowledge in patients. This work highlights several opportunities for future interventions to improve diagnosis messaging, starting with unifying how and when diagnosis messaging should occur. Once patients are fully informed of their CKD diagnosis, providers will be better enabled to work with patients in next steps of care to achieve best possible outcomes. 

## Funding 

This research was supported by an NIH NIDDK K23-DK097183 (JWN) grant. 

## Conflict of interest 

There are no conflicts of interest to report. 

**Table 1.** Questions and probes for interviews.

Questions	Probes
Do you typically tell patients with kidney disease that they have chronic kidney disease? If so, do you use these words, or other words? If yes, at what point in the diagnosis do you tell the patient?	Probes: As soon as you feel the diagnosis is confirmed, when you feel it will impact patient health, other.
Do you typically describe for patients the level of their disease severity?	Probes: For example, do you describe if they have mild, moderate, or severe disease? How do you discuss this with them, what specific information do you provide?
When do you think a person should be told they have kidney disease?	Probes: Never, as soon as it is identified, at the point it might impact health, near dialysis, whenever the doctor feels the patient is ready to hear the information, whenever the doctor wants to, other.
What components of a patient’s kidney disease diagnosis do you feel are difficult to explain to patients?	Probes: What makes it difficult? Do tools or resources could help make it less difficult?
What information do you feel patients need to enable implementation of most effective management of their chronic kidney disease?	Probes: Classes with patient peers, one-on-one peer supporter, brochures/written materials, trusted websites, an application for smart phones or electronic computer tablets, videos or audios, a health coach.
Research shows that some doctors are hesitant to tell patients they have “chronic kidney disease”. This is because some doctors feel it doesn’t change health management much. Do you feel it is important to tell a person they have chronic kidney disease?	Probe: Using the words “chronic kidney disease” or other words?
Do you/or your practice provide patients with resources outside of patient visits with the doctor, to learn more about kidney disease management?	Probes: Classes, brochures or other written materials or suggest website.

**Table 2.** Baseline characteristics of study population, self-reported.

Characteristic (N = 24)	Mean (SD) or n
Age (years)	46 (9)
Female	9
Race	
White	16
Asian/Asian-American	7
Other	1
Type of provider	
Primary-care provider	6
Nephrologist	18
Years in practice (i.e., years from medical school graduation)	22 (9)
Percent clinical time	51 (32)

**Table 3.** Provider perspectives on conveying a CKD diagnosis by discipline (PCP vs. nephrologist)*.

Tell patients they have CKD						
	Yes		Hesitant		Total	p-value
PCP	4	66.7%	2	33.3%	6	0.143
Nephrologists	17	94.4%	1	5.6%	18	
All providers	21	87.5%	3	12.5%	24	
Timing of informing patients they have CKD						
	Early		Depends		Total	p-value
PCP	2	40.0%	3	60.0%	5	0.063
Nephrologists	14	87.5%	2	12.5%	16	
All providers	16	76.2%	5	23.8%	21	
Language used to inform patients of CKD diagnosis						
	Used “CKD”		Used other terms		Total	p-value
PCP	0	0.0%	5	100.0%	5	0.110
Nephrologists	8	50.0%	8	50.0%	16	
All providers	8	38.1%	13	61.9%	21	

*Comparisons made using two-sided Fisher’s exact test. PCP = primary care provider; CKD = chronic kidney disease.
